# Supplementary figures and images for: Genetic reanalysis of patients with a difference of sex development carrying the NR5A1/SF-1 variant p.Gly146Ala has discovered other likely disease-causing variations
Source: PLoS One. 2023 Jul 11;18(7):e0287515. doi: 10.1371/journal.pone.0287515 (PMC10335684; doi:10.1371/journal.pone.0287515)

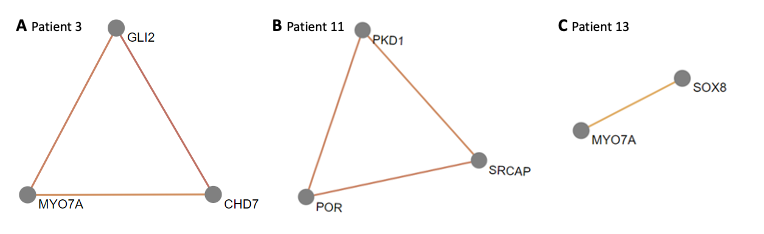

Supplement: S1 Fig — Networks were identified for patients 3, 11 and 13 respectively. To search for potential oligogenic disease networks, the Oligogenic Resource for Variant AnaLysis (ORVAL, https://orval.ibsquare.be/) was used. Nodes represent genes and edges connect two genes only, if between them there is at least one candidate disease-causing variant combination predicted by VarCoPP. The colour of the edge represents the pathogenicity score for that pair of genes. This score is represented in a colour range from brown (higher pathogenicity score) to yellow (lower pathogenicity score). (DOCX) [file pone.0287515.s001.docx]
