# Supplementary material for: Genetic reanalysis of patients with a difference of sex development carrying the NR5A1/SF-1 variant p.Gly146Ala has discovered other likely disease-causing variations
Source: PLoS One. 2023 Jul 11;18(7):e0287515. doi: 10.1371/journal.pone.0287515 (PMC10335684; doi:10.1371/journal.pone.0287515)
Supplement: S1 Table — CHH, central causes of hypogonadism; G det, gonadal determination; G diff, gonadal differentiation. (DOCX) [file pone.0287515.s002.docx]

| **Gene (*locus*)** | **Alias** | **Transcript** | **Role in DSD** |
| --- | --- | --- | --- |
| *AMH* (19p13.3) | Anti-Mullerian Hormone | NM_000479.3 | G diff |
| *AMHR2* (12q13.13) | Anti-Mullerian Hormone Receptor Type 2 | NM_020547.2 | G diff |
| *AR* (Xq12) | Androgen Receptor | NM_000044.3 | G diff |
| *ATRX* (Xq21.1) | ATP-Dependent Helicase ATRX | NM_000489.4 | G diff |
| *BMP15* (Xp11.22) | Bone Morphogenetic Protein 15 | NM_005448.2 | G det |
| *CBX2* (17q25.3) | Chromobox homolog 2 | NM_005189.2 | G det |
| *CYP11A1* (15q24.1) | Cytochrome P450 Family 11 Subfamily A Member 1 | NM_000781.2 | G diff |
| *CYP11B1* (8q24.3) | Cytochrome P450 Family 11 Subfamily B Member 1 | NM_000497.3 | G diff |
| *CYP17A1* (10q24.32) | Cytochrome P450 Family 17 Subfamily A Member 1 | NM_000102.3 | G diff |
| *CYP19A1* (15q21.2) | Cytochrome P450 Family 19 Subfamily A Member 1 | NM_000103.3 | G diff |
| *CYP21A2* (6p21.33) | Cytochrome P450 Family 21 Subfamily A Member 2 | NM_000500.7 | G diff |
| *DHH* (12q13.12) | Desert Hedgehog | NM_021044.2 | G det |
| *DMRT1* (9p24.3) | Doublesex And Mab-3 Related Transcription Factor 1 | NM_021951.2 | G det |
| *DMRT2* (9p24.3) | Doublesex And Mab-3 Related Transcription Factor 2 | NM_181872.4 | G Dev |
| *ESR1* (6q25.1-q25.2) | Oestrogen Receptor 1, Nuclear Receptor Subfamily 3 Group A Member 1 | NM_001122740.1 | G diff |
| *ESR2* (14q23.2-q23.3) | Oestrogen Receptor 2, Nuclear Receptor Subfamily 3 Group A Member 2 | NM_001437.2 | G diff |
| *FGF9* (13q12.11) | Fibroblast Growth Factor 9 | NM_002010.2 | G det |
| *FOXL2* (3q22.3) | Forkhead Box L2 | NM_023067.3 | G det |
| *FOXO3* (6q21) | Forkhead Box O3 | NM_001455.3 | G det |
| *FSHR* (2p16.3) | Follicle Stimulating Hormone Receptor | NM_000145.3 | CHH |
| *GATA4* (8p23.1) | GATA Binding Protein 4 | NM_002052.3 | G det |
| *HARS2 (5q31.3)* | Histidyl-TRNA Synthetase 2, Mitochondrial | NM_012208.3 | G diff |
| *HSD17B3 (*9q22.32) | Hydroxysteroid 17-Beta Dehydrogenase 3 | NM_000197.1 | G diff |
| *HSD17B4* (5q23.1) | Hydroxysteroid 17-Beta Dehydrogenase 4 | NM_000414.3 | G diff |
| *HSD3B2* (1p12) | Hydroxy-Delta-5-Steroid Dehydrogenase, 3 Beta and Steroid Delta-Isomerase 2 | NM_000198.3 | G diff |
| *INHA* (2q35) | Inhibin Subunit Alpha | NM_002191.3 | G det |
| *INSL3* (19p13.11) | Insulin Like 3 | NM_001265587.1 | G diff |
| *KISS1* (1q32.1) | Kisspeptin-1 | NM_002256.3 | CHH |
| *KISS1R* (19p13.3) | KISS1 Receptor | NM_032551.4 | CHH |
| *LHCGR* (2p16.3) | Luteinizing Hormone/Choriogonadotropin Receptor | NM_000233.3 | G diff |
| *MAMLD1* (Xq28) | Mastermind Like Domain Containing 1, CXorf6 | NM_001177465.2 | G det |
| *MAP3K1* (5q11.2) | Mitogen-Activated Protein Kinase Kinase Kinase 1 | NM_005921.1 | G det |
| *NR0B1* (Xp21.2) | Nuclear Receptor Subfamily 0 Group B Member 1 | NM_000475.4 | G det |
| *NR5A1* (9q33.3) | Nuclear Receptor Subfamily 5 Group A Member 1 | NM_004959.4 | G det |
| *POR* (7q11.23) | Cytochrome P450 Oxidoreductase | NM_000941.2 | G diff |
| *PSMC3IP* (17q21.2) | Proteasome 26S ATPase Subunit 3-Interacting Protein | NM_016556.3 | G det |
| *RSPO1* (1p34.3) | R-Spondin 1 | NM_001242908.1 | G det |
| *RXFP2* (13q13.1) | Relaxin Family Peptide Receptor 2, GREAT, LGR8 | NM_130806.3 | G diff |
| *SOX3* (Xq27.1) | SRY (Sex Determining Region Y)-Box 3 | NM_005634.2 | G det |
| *SOX9* (17q24.3) | SRY (Sex Determining Region Y)-Box 9 | NM_000346.3 | G det |
| *SRD5A2* (2p23.1) | Steroid 5 Alpha-Reductase 2 | NM_000348.3 | G diff |
| *SRY* (Yp11.2) | Sex Determining Region Y | NM_003140.2 | G det |
| *STAR* (8p11.23) | Steroidogenic Acute Regulator | NM_000349.2 | G diff |
| *TSPYL1* (6q22.1) | Testis-Specific Y-Encoded-Like Protein 1 | NM_003309.3 | G det |
| *WNT4* (1p36.12) | Wingless-Type MMTV Integration Site Family, Member 4 | NM_030761.4 | G det |
| *WT1* (11p13) | Wilms Tumour 1 | NM_024426.4 | G det |
| *WWOX* (16q23.1-q23.2) | WW Domain Containing Oxidoreductase | NM_016373.2 | G det |
| *ZFPM2* (8q23.1) | Zinc Finger Protein, FOG Family Member 2, Friend Of GATA 2 | NM_012082.3 | G det |
