# Supplementary material for: Genetic reanalysis of patients with a difference of sex development carrying the NR5A1/SF-1 variant p.Gly146Ala has discovered other likely disease-causing variations
Source: PLoS One. 2023 Jul 11;18(7):e0287515. doi: 10.1371/journal.pone.0287515 (PMC10335684; doi:10.1371/journal.pone.0287515)
Supplement: S2 Table — ACTH, adrenocorticotropic hormone; AMH, anti-Müllerian hormone; d, days; DHEA-S, dehydroepiandrosterone sulfate; DHT, dihydrotestosterone; E2, estradiol; FSH, follicle-stimulating hormone; LH, luteinizing hormone; mo, month; N, normal; ND, not determined; PRL, prolactin; P4, progesterone; Y, years; Δ4-A, delta 4-androstenedione; 17OHP4, 17-hydroxy-progesterone. (*) Values after stimulation with hCG or ACTH. Out of range values for karyotypic sex and age are given in bold. (DOCX) [file pone.0287515.s003.docx]

| **Patient** | **Phenotype** | **Age at evaluation** | **Adrenal steroidogenesis** | | | | | **Gonadal function** | | | | | | |
| --- | --- | --- | --- | --- | --- | --- | --- | --- | --- | --- | --- | --- | --- | --- |
|  |  |  | **ACTH (pg/mL)** | **Cortisol (µg/dL)** | **17OHP4 (ng/mL)** | **DHEA-S (ng/mL)** | **Δ4-A (ng/mL)** | **Testosterone (ng/dL)** | **FSH (U/L)** | **LH (U/L)** | **E2 (pg/mL)** | **DHT (ng/ml)** | | **AMH (ng/mL)** |
| 1 | 15y, after treatment, mature penis and scrotum, pubarche IV | At birth | <25 |  |  |  |  | **34.7** |  |  |  |  | |  |
| 2 | 6y, micropenis, scrotal hypospadias, bifid scrotum. | 6y |  |  | **<0.1** | <180 |  | <10/44.1* |  |  |  | **0.3/**0.1* | |  |
| 3 | 9y, micropenis (2cm), scrotal hypospadias. | 9y |  |  |  |  |  | 80* |  |  |  | N* | |  |
| 4 | 6y, curved penis (2cm), scrotal hypospadias, bifid scrotum, right inguinal hernia. | 6y |  |  | 0.2 | <180 | <0.3 | <10/125* | <1.5 | <1.5 |  | <0.1/0.1* | |  |
| 5 | 3y, rudimentary penis (<0.5cm), pubarche I. | 4y |  |  |  |  | **0.5/<0.3*** | **14/59*** | 1,3 | <0.1 |  | **0.1/0.1*** | | 8.8 |
| 6 | 2y, curved penis, scrotal hypospadias. | 2y |  | 17.7 |  | <150 | <0.3/<0.3* | <10/110.4* |  |  |  | **<0.1/<0.1*** | |  |
| 7 | 6y, curved penis (4.4-4.5cm), scrotal hypospadias, cryptorchidism and bifid scrotum | 6y |  | 11 | 0.4 | 270 | <0.3/<0.3* | <10/263.4* | 0.8 | <0.1 |  | 0.1/0.3* | | 22.8 |
| 8 | 7y, right inguinal hernia. At puberty, primary amenorrhea, ovarian and uterine agenesis. 24y, left inguinal hernia. 35y, female external genitalia, thelarche II-III, erectile organ is buried, one opening for urethra and vagina. | 35y | 27.9 | 21.3 | 0.2 | 1390 | 1.3 | **19** | **67.0** | **48.5** | 5 |  | |  |
| 9 | 3y, curved penis, scrotal hypospadias, atrophic scrotum, non-palpable testes. | 7y |  |  | 0.3 | 1100 |  | <20 | 0.5 | <10 |  |  | |  |
| 10 | 11mo, distal hypospadias, surgery. 12y, after treatment, penis (5.2cm) buried in fat, testes 10-12ml, pubarche III. | 3y |  | 13.9 | 0.1 | <150 | <0.3/<0.3* | **<10/**72.8* | **3** | <0.1 |  | **<0.1** | | 7 |
| 11 | At birth, left scrotal hypoplasia. 4y, orchidopexy. 11y, penis 3.5-4cm, testes 2-4ml, gynecomastia, pubarche I. | 10y |  |  |  |  | <0.3 | <10 | 1.3 | <0.1 |  | 0.1 | | 193 |
| 12 | 15y, primary amenorrhea. 44y, absence of developed breast, pubic and axillary hair. | ND |  |  |  |  |  |  |  |  |  |  |  | |
| 13 | 7d, micropenis, fused labia minora, non-palpable gonads, tight vagina. | 15d |  | 21.4 | **8.2** | 247 | **3.2** | 1.5 | **17.3** | **17.1** | 16.2 |  |  | |
