# Supplementary material for: Genetic reanalysis of patients with a difference of sex development carrying the NR5A1/SF-1 variant p.Gly146Ala has discovered other likely disease-causing variations
Source: PLoS One. 2023 Jul 11;18(7):e0287515. doi: 10.1371/journal.pone.0287515 (PMC10335684; doi:10.1371/journal.pone.0287515)
Supplement: S3 Table — B, benign; Dam, damaging; DC, disease causing; Dis, disease; LB, likely benign; LP, likely pathogenic; N, neutral; ND, not determined; P, polymorphism; Path, pathogenic; Psdam, possibly damaging; Prben, probably benign; Prdam, probably damaging; VUS, variant of unknown significance. aSpecific allele frequency for the origin and karyotype of the patient. bCADD phred score >20 indicates that the variant is predicted to be the 1% most deleterious substitution that you can do to the human genome. For each gene, sequence information is based on: ADAMTS16 (NM_139056.4), AR (NM_000044.3), CHD7 (NM_017780.4), COL27A1 (NM_032888.4), FGFR3 (NM_000142.5), GLI2 (NM_001374353.1), INSR (NM_000208.4), LHCGR (NM_000233.4), MYO7A (NM_000260.4), NRP1 (NM_003873.7), PKD1 (NM_001009944.3), POR (NM_001395413.1), SOX8 (NM_014587.5), SOX9 (NM_000346.4), SRCAP (NM_006662.3), TYRO3 (NM_006293.4) and VDR (NM_000376.3). (DOCX) [file pone.0287515.s004.docx]

| **Patient** | **Gene** | **Variant** | **Exon** | **GnomAD (Overall/Specific^a^)** | **ClinVar** | **ACMG classification (Criteria)** | **SIFT** | **Provean** | **Polyphen** | **Mutation Taster** | **Panther** | **SNPs and Go** | **M-CAP** | **CADD^b^** | **REVEL** |
| --- | --- | --- | --- | --- | --- | --- | --- | --- | --- | --- | --- | --- | --- | --- | --- |
| 1 | *FGFR3* | p.Cys545Hisfs*17 | 12 | ND/ND | ND | LP (PVS1,PM2) | ND | ND | ND | ND | ND | ND | ND | ND | ND |
|  | *ADAMTS16* | p.His608* | 12 | ND/ND | ND | LP (PVS1,PM2) | ND | ND | ND | ND | ND | ND | ND | ND | ND |
|  | *INSR* | p.Pro220Hisfs*4 | 3 | ND/ND | ND | LP (PVS1,PM2) | ND | ND | ND | ND | ND | ND | ND | ND | ND |
| 3 | *GLI2* | p.Gln1176His | 13 | 0.001603/0.004818 | B | B (BP6,BS1,BS2,BP4,BP1) | Path | VUS | Prdam | VUS | Prdam | Dis | B | 22.5 | B |
|  | *CHD7* | p.His541Gln | 2 | 0.0001314/0.0001554 | ND | VUS (PM2,PP3) | B | B | B | VUS | ND | Dis | VUS | ND | B |
|  | *MYO7A* | p.Gly961Asp | 23 | 0.0005718/0.002384 | VUS | VUS (PM2) | B | Path | Psdam | VUS | ND | Dis | VUS | 26.4 | VUS |
|  | *VDR* | p.Thr59Ile | 4 | 0.0006569/0.002487 | LB | VUS (PM1,PM2,PP3,BP6) | B | VUS | Psdam | VUS | Prdam | Dis | VUS | 24.8 | VUS |
| 6 | *NRP1* | p.Pro61Gln | 2 | ND/ND | ND | LB (BP1,BP4,PM2) | Path | VUS | Prdam | VUS | Psdam | Dis | B | ND | VUS |
| 8 | *LHCGR* | p.Ser253Pro | 9 | ND/ND | ND | VUS (PM2) | VUS | VUS | Prdam | VUS | Psdam | ND | VUS | ND | VUS |
| 9 | *COL27A1* | c.3645_3651+5del | 37 | 0.001025/0.002889 | LB | VUS (PP3,PM2,BP6) | VUS | Path | Prdam | B | Prdam | N | B | 33 | B |
|  | *TYRO3* | p.Ala223HisfsTer21 | 5 | ND/ND | ND | VUS (PM2) | ND | ND | ND | ND | ND | ND | ND | ND | ND |
| 10 | *SOX8* | p.Thr226Pro | 3 | 0.0003858/0.0007298 | ND | VUS (PP3,PM2,BP1) | B | Path | Prdam | VUS | Prdam | Dis | Path | ND | Path |
| 11 | *POR* | p.Thr560Met | 14 | 0.00005255/0.0006859 | ND | VUS (PM2) | B | VUS | B | VUS | Prdam | N | Path | 24.4 | Path |
|  | *PKD1* | p.Pro875Leu | 11 | ND/ND | ND | VUS (PM2,PP3) | Path | Path | Prdam | VUS | ND | Dis | Path | ND | VUS |
|  | *SRCAP* | p.Arg2381His | 34 | 0.000006576/0.000 | ND | VUS (PM2,BP1) | Path | B | Prdam | B | Prben | N | VUS | 26.8 | B |
|  | *SOX9* | p.Pro238Thrfs*14 | 3 | ND/ND | VUS | LP (PVS1) | ND | ND | ND | ND | ND | ND | ND | ND | ND |
| 12 | *AR* | p.Arg775Cys | 6 | ND/ND | P | P (PP5,PS3,PP3,PM1) | Path | Path | ND | VUS | Prdam | ND | Path | 28.2 | Path |
| 13 | *MYO7A* | p.Arg1420His | 32 | 0.00001971/0.000 | VUS | VUS (PM2) | B | B | B | VUS | ND | Dis | VUS | 25.6 | VUS |
|  | *SOX8* | p.Lys232Gln | 3 | 0.0002685/0.0002686 | ND | VUS (PP3,PM2,BP1) | VUS | VUS | Prdam | VUS | Prdam | Dis | Path | 27.5 | Path |
