# Supplementary material for: Genetic reanalysis of patients with a difference of sex development carrying the NR5A1/SF-1 variant p.Gly146Ala has discovered other likely disease-causing variations
Source: PLoS One. 2023 Jul 11;18(7):e0287515. doi: 10.1371/journal.pone.0287515 (PMC10335684; doi:10.1371/journal.pone.0287515)
Supplement: S4 Table — Variants were discarded after filtering due to weak relation to DSD, zygosity or absence of correspondence to the phenotype. B, benign; Het, heterozygous; Hom, homozygous; LB, likely benign; LP, likely pathogenic; ND, not determined; P, pathogenic; G6PDH, glucose-6-phosphate dehydrogenase; VUS, variant of unknown significance. For each gene, sequence information is based on: ADCY7 (NM_001114.5), AMH (NM_000479.5), ARVCF (NM_001670.3), ATM (NM_000051.4), ATR (NM_001184.4), BBS5 (NM_152384.3), CDH1 (NM_004360.5), CEBPB (NM_005194.4), COL9A3 (NM_001853.4), DHCR24 (NM_014762.4), EXO1 (NM_130398.4), FOXO3 (NM_001455.4), G6PD (NM_001360016.2), GEMIN4 (NM_015721.3), GHR (NM_000163.5), GPR83 (NM_016540.4), GRIN2C (NM_000835.6), HFE (NM_000410.4), IFFO1 (NM_001193457.2), IL6ST (NM_002184.4), INPP5F (NM_014937.4), ITIH3 (NM_002217.4), KYAT3 (NM_001008661.3), MKS1 (NM_017777.4), MTRR (NM_002454.3), NBN (NM_002485.5), NCOA3 (NM_181659.3), NF1 (NM_001042492.3), NOBOX (NM_001080413.3), POLG (NM_002693.3), POLM (NM_013284.4), PPIL2 (NM_014337.4), ROS1 (NM_001378902.1), SARDH (NM_001134707.2), STAG3 (NM_001282717.2), THBD (NM_000361.3), UBR2 (NM_001363705.2), VPS18 (NM_020857.3) and ZMIZ2 (NM_031449.4). (DOCX) [file pone.0287515.s005.docx]

| **Patient** | **Chromosome position** | **Gene (Name)** | **Variant** | **dbSNP** | **Zygosity** | **ACMG classification** | **Previously reported** |
| --- | --- | --- | --- | --- | --- | --- | --- |
| 1 | 3:52834677 | *ITIH3* (Inter-Alpha-Trypsin Inhibitor Heavy Chain 3) | c.1199_1200del;p.Val400Glyfs*2 | ND | het | LP | No |
|  | 5:7886651 | *MTRR* (5-Methyltetrahydrofolate-Homocysteine Methyltransferase Reductase) | c.1094C>A;p.Ser365Tyr | rs1293804430 | het | VUS | No |
|  | 7:44112925 | *POLM* (DNA Polymerase Mu) | c.1450dup;p.Leu484Profs*2 | ND | het | VUS | No |
|  | 7:44801187 | *ZMIZ2* (Zinc Finger MIZ-Type Containing 2) | c.1381dup;p.Met461Asnfs*4 | ND | het | LP | No |
|  | 8:89982837 | *NBN* (Nibrin) | c.56T>G;p.Leu19Trp | rs749263651 | het | VUS | No |
|  | 9:133732566 | *SARDH* (Sarcosine Dehydrogenase) | c.367G>T;p.Val123Leu | rs1391072810 | het | VUS | No |
|  | 15:41191433 | *VPS18* (VPS18 Core Subunit Of CORVET And HOPS Complexes) | c.417_418insA;p.Gln140Thrfs*32 | ND | hom | VUS | No |
|  | 16:50334767 | *ADCY7* (Adenylate Cyclase 7) | c.1218_1219del;p.Glu406Aspfs*98 | ND | het | LP | No |
|  | 17:31169974 | *NF1* (Neurofibromin 1) | c.563C>A;p.Ala188Glu | ND | het | VUS | No |
|  | 22:19968739 | *ARVCF* (ARVCF Delta Catenin Family Member) | c.889_890insG;p.His297Argfs*11 | ND | hom | VUS | No |
| 2 | 1:88962138 | *KYAT3* (Kynurenine Aminotransferase 3) | c.461T>C;p.Leu154Pro | rs75696718 | het | B | No |
|  | 3:142553920 | *ATR* (ATR Serine/Threonine Kinase) | c.2437A>G;p.Met813Val | rs769648140 | het | VUS | No |
|  | 5:55960477 | *IL6ST* (Interleukin 6 Cytokine Family Signal Transducer) | c.898C>T;p.Arg300Cys | rs141500365 | het | LB | No |
|  | 16:68819395 | *CDH1* (Cadherin 1) | c.1681T>C;p.Tyr561His | ND | het | VUS | No |
|  | 20:50192055 | *CEBPB* (CCAAT Enhancer Binding Protein Beta) | c.1022C>T;p.Ser341Phe | ND | het | LB | No |
| 4 | 1:88962138 | *KYAT3* (Kynurenine Aminotransferase 3) | c.461T>C;p.Leu154Pro | rs75696718 | het | B |  |
|  | 6:117394711 | *ROS1* (ROS Proto-Oncogene 1, Receptor Tyrosine Kinase) | c.911del;p.Leu304Tyrfs*7 | rs763595603 | het | LP | No |
|  | X:154532945 | *G6PD* (Glucose-6-Phosphate Dehydrogenase) | c.1048G>C;p.Asp350His | rs34193178 | het | LB | G6PDH deficiency (1) |
| 6 | 3:142553920 | *ATR* (ATR Serine/Threonine Kinase) | c.2437A>G;p.Met813Val | rs769648140 | het | VUS | No |
|  | 17:74842561 | *GRIN2C* ( Glutamate Ionotropic Receptor NMDA Type Subunit 2C) | c.3575_3576del;p.Leu1192Argfs*86 | ND | het | VUS | No |
| 7 | 1:241857358 | *EXO1* (Exonuclease 1) | c.419_420del;p.Gln140Argfs*10 | rs1491146265 | het | LP | No |
|  | 6:108663973 | *FOXO3* (Forkhead Box O3) | c.1143_1144insG;p.Leu382Alafs*3 | rs758436116 | het | LP | No |
|  | 6:26087507 | *HFE* (Homeostatic Iron Regulator) | c.67C>T;p.Arg23Cys | rs761203501 | het | VUS | No |
|  | 12:6555481 | *IFFO1* (Intermediate Filament Family Orphan 1) | c.538_549del;p.Ser180_Thr183del | rs756975788 | het | VUS | No |
| 9 | 5:42718493 | *GHR* (Growth Hormone Receptor) | c.986A>G;p.His329Arg | rs775435127 | het | VUS | No |
|  | 6:42684814 | *UBR2* (Ubiquitin Protein Ligase E3 Component N-Recognin 2) | c.4796A>C;p.Lys1599Thr | rs1044454747 | het | VUS | No |
|  | 7:100211095 | *STAG3* (Stromal Antigen 3) | c.3323T>C;p.Leu1108Pro | ND | het | VUS | No |
|  | 10:119827427 | *INPP5F* (Inositol Polyphosphate-5-Phosphatase F) | c.3046G>T;p.Val1016Phe | rs150134182 | het | VUS | No |
|  | 11:94396457 | *GPR83* (G Protein-Coupled Receptor 83) | c.455A>G;p.Tyr152Cys | rs1234084993 | het | VUS | No |
|  | 15:89325562 | *POLG* (DNA Polymerase Gamma, Catalytic Subunit) | c.1837C>T;p.His613Tyr | rs147407423 | het | VUS | Ptosis, mypopathy and severe cerebellar atrophy (2) |
|  | 19:2249369 | *AMH* (Anti-Mullerian Hormone) | c.37C>G;p.Leu13Val | rs754607106 | het | VUS | No |
|  | 20:23048319 | *THBD* (Thrombomodulin) | c.1186C>A;p.Pro396Thr | ND | het | VUS | No |
|  | 20:47649093 | *NCOA3* (Nuclear Receptor Coactivator 3) | c.3645_3651+5del | rs770158269 | het | LP | No |
| 10 | 5:42718663 | *GHR* (Growth Hormone Receptor) | c.1156C>T;p.Arg386Cys | rs34853905 | het | VUS | No |
|  | 7:144399832 | *NOBOX* (NOBOX Oogenesis Homeobox) | c.1079G>A;p.Arg360Gln | rs199538689 | het | LB | No |
|  | 11:108335849 | *ATM* (ATM Serine/Threonine Kinase) | c.8156G>A;p.Arg2719His | rs55982963 | het | VUS | Susceptibility to breast cancer (3) |
|  | 17:58218727 | *MKS1* (MKS Transition Zone Complex Subunit 1) | c.83T>C;p.Val28Ala | rs201957874 | het | VUS | No |
|  | 17:58212983 | *MKS1* (MKS Transition Zone Complex Subunit 1) | c.857A>G;p.Asp286Gly | rs151023718 | het | VUS | Bardet-Biedl (4) |
|  | 17:746992 | *GEMIN4* ( Gem Nuclear Organelle Associated Protein 4) | c.1050_1051del;p.Asp350Glufs*28 | rs758240351 | het | VUS | No |
| 11 | 1:54886909 | *DHCR24* (24-Dehydrocholesterol Reductase) | c.211G>T;p.Val71Leu | ND | het | VUS | No |
|  | 2:169493760 | *BBS5* (Bardet-Biedl Syndrome 5) | c.542T>C;p.Phe181Ser | rs758508869 | het | VUS | No |
|  | 5:42718990 | *GHR* (Growth Hormone Receptor) | c.1483C>A;p.Pro495Thr | rs6183 | het | P | Susceptibility to lung cancer (5) |
|  | 20:62837104 | *COL9A3* (Collagen Type IX Alpha 3 Chain) | c.1625C>T;p.Ala542Val | rs753247678 | het | VUS | No |
|  | 22:21683186 | *PPIL2* (Peptidylprolyl Isomerase Like 2) | c.482C>T;p.Pro161Leu | rs765685353 | het | VUS | No |

1. Bulliamy T, Luzzatto L, Hirono A, Beutler E. Hematologically important mutations: glucose-6-phosphate dehydrogenase. Blood Cells Mol Dis. 1997;23(2):302-13.

2. Da Pozzo P, Cardaioli E, Rubegni A, Gallus GN, Malandrini A, Rufa A, et al. Novel POLG mutations and variable clinical phenotypes in 13 Italian patients. Neurol Sci. 2017;38(4):563-70.

3. Maxwell KN, Wubbenhorst B, D'Andrea K, Garman B, Long JM, Powers J, et al. Prevalence of mutations in a panel of breast cancer susceptibility genes in BRCA1/2-negative patients with early-onset breast cancer. Genetics in medicine : official journal of the American College of Medical Genetics. 2015;17(8):630-8.

4. Leitch CC, Zaghloul NA, Davis EE, Stoetzel C, Diaz-Font A, Rix S, et al. Hypomorphic mutations in syndromic encephalocele genes are associated with Bardet-Biedl syndrome. Nature genetics. 2008;40(4):443-8.

5. Rudd MF, Webb EL, Matakidou A, Sellick GS, Williams RD, Bridle H, et al. Variants in the GH-IGF axis confer susceptibility to lung cancer. Genome Res. 2006;16(6):693-701.
